# Serum 25-hydroxyvitamin D_3_ is associated with advanced glycation end products (AGEs) measured as skin autofluorescence: The Rotterdam Study

**DOI:** 10.1007/s10654-018-0444-2

**Published:** 2018-09-25

**Authors:** Jinluan Chen, Daniel van der Duin, Natalia Campos-Obando, Mohammad Arfan Ikram, Tamar E. C. Nijsten, André G. Uitterlinden, Maria Carola Zillikens

**Affiliations:** 1 Department of Internal Medicine, Erasmus MC, University Medical Center Rotterdam, Rotterdam, South Holland The Netherlands; 2 Department of Epidemiology, Erasmus MC, University Medical Center Rotterdam, Rotterdam, South Holland The Netherlands; 3 Department of Dermatology, Erasmus MC, University Medical Center Rotterdam, Rotterdam, South Holland The Netherlands

**Keywords:** Skin autofluorescence, Vitamin D, Aging, Diabetes, Coffee, Smoking

## Abstract

Advanced glycation end products (AGEs) accumulate in tissues with aging and may influence age-related diseases. They can be estimated non-invasively by skin autofluorescence (SAF) using the AGE Reader™. Serum 25-hydroxyvitamin D_3_ (25(OH)D_3_) may inhibit AGEs accumulation through anti-oxidative and anti-inflammatory properties but evidence in humans is scarce. The objective was to investigate the association between serum 25(OH)D_3_ and SAF in the population-based cohort study. Serum 25(OH)D_3_ and other covariates were measured at baseline. SAF was measured on average 11.5 years later. Known risk factors for AGE accumulation such as higher age, BMI, and coffee intake, male sex, smoking, diabetes, and decreased renal function were measured at baseline. Linear regression models were adopted to explore the association between 25(OH)D_3_ and SAF with adjustment for confounders. Interaction terms were tested to identify effect modification. The study was conducted in the general community. 2746 community-dwelling participants (age ≥ 45 years) from the Rotterdam Study were included. Serum 25(OH)D_3_ inversely associated with SAF and explained 1.5% of the variance (unstandardized B = − 0.002 (95% CI[− 0.003, − 0.002]), standardized β = − 0.125), independently of known risk factors and medication intake. The association was present in both diabetics (B = − 0.004 (95% CI[− 0.008, − 0.001]), β = − 0.192) and non-diabetics (B = − 0.002 (95% CI[− 0.003, − 0.002]), β = − 0.122), both sexes, both smokers and non-smokers and in each RS subcohort. Serum 25(OH)D_3_ concentration was significantly and inversely associated with SAF measured prospectively, also after adjustment for known risk factors for high SAF and the number of medication used, but the causal chain is yet to be explored in future studies.

*Clinical Trial Registry* (1) Netherlands National Trial Register: Trial ID: NTR6831 (http://www.trialregister.nl/trialreg/admin/rctview.asp?TC=6831). (2) WHO International Clinical Trials Registry Platform: under shared catalogue number NTR6831 (www.who.int/ictrp/network/primary/en/).

**Electronic supplementary material:**

The online version of this article (10.1007/s10654-018-0444-2) contains supplementary material, which is available to authorized users.

## Introduction

Advanced glycation end products (AGEs) are a heterogeneous group of highly oxidized end products formed through non-enzymatic attachment of sugars to free amino groups of proteins, lipids and nucleic acids [1, 2]. AGEs accumulate in long-lived tissues with a half-life time of many years [3]; their formation is irreversible and they are difficult to be degraded and cleared from the tissues. Endogenous accumulation of AGEs is accelerated under conditions of hyperglycemia, high oxidative stress and chronic inflammation [4]. Exogenous factors such as dietary AGEs [5] and smoking-induced oxidative stress and inhaled glycotoxins that are absorbed into the bloodstream through the alveoli [6] also contribute to the amount of AGEs in vivo. A decrease in renal filtration rate lowers the clearance rate of AGEs and can thereby contribute to their accumulation [7].

AGEs accumulation contributes to the aging process [8] and age-related diseases [4] through formation of covalent crosslinking of proteins in the extracellular matrix such as collagen and elastin which lead to tissue stiffening, influencing molecules’ functions by binding to active sites of molecules, and inducing inflammation and cellular dysfunction through interaction with the receptor for AGEs [4, 9]. They may also have genotoxic effects [10]. Aside from the involvement in diabetic complications such as renal insufficiency and neuropathy [11], AGEs have been implicated in cardiovascular disease [12], bone pathology [13], lung diseases [14], neurodegenerative diseases [15] and some types of cancer [16].

Vitamin D and its analogs have been reported to exert anti-inflammation effect, which could influence AGE formation [17]. They were reported to prevent AGEs formation on rat cardiovascular tissue [18] and increase serum sRAGE level which plays a role in the clearance of AGEs as reported in human studies [19]. These studies led to the hypothesis that vitamin D can counteract AGEs accumulation but evidence for such a relation is currently still scarce. Firstly, the laboratory studies were restricted to particular cell types of short study duration and need cross validation. Secondly, existing human studies were small-scaled, cross-sectional, limited to special patient groups, and conclusions were contradictory. In a study of 276 type 1 and type 2 diabetics and 121 non-diabetic controls, no association was observed between 25(OH)D_3_ and SAF [20]. In contrast, a significant inverse relationship between 25(OH)D_3_ and AGEs measured by skin autofluorescence (SAF) was observed in a recent study of 245 type 2 diabetes patients treated with lifestyle advice, metformin and/or sulphonylurea-derivatives [21]. Evidence from large population based, prospective studies in the general population is lacking.

A fluorescence method has been developed to assess the accumulation of AGEs in the human skin non-invasively and quickly by the use of “AGE Reader™” through measuring specific wavelength scope of skin autofluorescence (SAF) emitted after being excited by specific wavelength scope of light. The fluorescent property of AGEs has already been utilized to measure AGEs in skin biopsies and homogenates [22]. Within the Rotterdam study, skin AGEs have been recently measured with the “AGE Reader™” as SAF. SAF has been reported in various publications to have a positive association with diabetes complications and other diseases [23]. The goal of this study was to investigate the independent association between serum 25(OH)D_3_ concentration at baseline and SAF assessed 11.5 years later in a large and densely phenotyped population-based cohort.

## Subjects and methods

### Study population

Participants were recruited from three subcohorts of the Rotterdam Study, a population-based prospective cohort study. It was initiated in 1990, when inhabitants of the suburb Ommoord in the city of Rotterdam were invited to participate. The first subcohort RS-I started in 1990, including n = 7983 participants of 55 years and over. In 2000 a second subcohort (RS-II) started with n = 3011 participants aged 55 years and over. The third subcohort(RS-III) including n = 3932 participants of 45 years and older started in 2006. All participants were examined at baseline and every 3–5 years follow-up examinations have been taking place. The design and objectives of the Rotterdam Study have been extensively described previously [24]. The Rotterdam Study was approved by the institutional review board (Medical ethics Committee) of the Erasmus Medical center and by the review board of The Netherlands Ministry of health, Welfare and Sports. All participants in the present analysis provided written informed consent to participate.

**Fig. 1:**
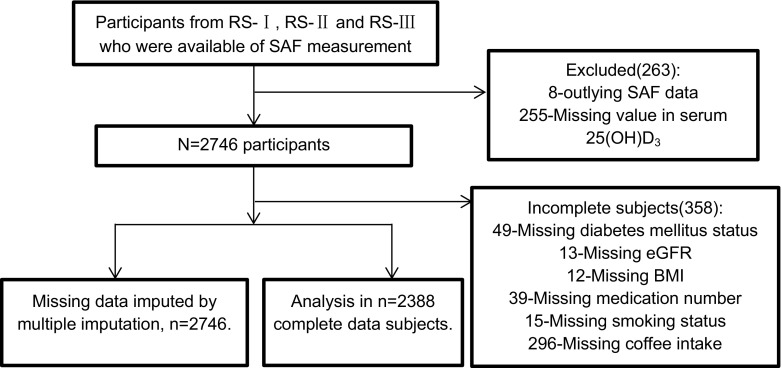
Flowchart of participant selection from the Rotterdam Study for the analysis in this study. Abbreviations: *SAF* skin auto-fluorescence; *BMI* body mass index; 25(OH)D_3_, 25-Hydroxyvitamin D_3_; *eGFR* estimated glomerular filtration rate calculated by the CKD-EPI

The AGE Reader™ was introduced in the Rotterdam Study to measure skin autofluorescence (SAF) in 2013, so far 3009 participants (754 in RS-I 6th follow-up, 1088 in RS-II 4th follow-up and 1167 in RS-III 2nd follow-up) have SAF measurements available. Values were defined as outliers in SAF and excluded from the analysis if it exceed the scope of mean ± 4SD, based on this 8 participants were excluded. 255 participants with missing value in serum 25(OH)D_3_ concentration were additionally excluded mainly because of inadequate blood sample volume for analysis, leaving 2746 participants included in the study. Missing value in other covariates was subsequently checked, identifying 358 participants with incomplete data and the other 2388 participants with full record. The inclusion and exclusion of participants in this study are shown in Fig. 1.

### Measurement of serum 25(OH)D_3_ concentration at baseline

Blood samples were collected at baseline of this research in RS-I 3rd follow-up, RS-II 1st follow-up and RS-III 1st visit. Serum 25(OH)D_3_ was assessed using electrochemiluminescence immunoassay (COBAS vitamin D total assay reagent, Roche Diagnostics GmbH, Germany) measured by MODULAR ANALYTICS, Elecsys or Cobas e immunoassay analyzer (Roche Diagnostics GmbH, Germany). The test range was between 7.5 and 175 nmol L^−1^ with a functional sensitivity of 10 nmol L^−1^. The intermediate precision of the test was CV < 13.1% and within-run precision was CV < 7.8%. The blood samples were collected at each visit and stored in the freezer at − 80 °C until they were measured for 25(OH)D_3_ together by the same techniques, instruments and tool kits in the same time period. The concentration of 25(OH)D_3_ has been adjusted for seasonal variance using cosinor analysis [25].

### Assessment of covariates at baseline

All covariates were obtained at baseline when serum 25(OH)D_3_ was measured. Age was calculated by using the date of birth and blood collection date. Diabetic participants were defined as having fasting blood glucose level ≥ 7.0 mmol L^−1^, or using anti-diabetes medication or reporting to have been diagnosed with diabetes. Smoking status was categorized as never, past or current smoker based on their smoking history of cigarette, cigar and pipe information during home interview. The anthropometric characteristics weight and height were measured at the research center without shoes and with light clothes, BMI (kg/m^2^) was calculated as weight divided by height^2^. Blood was drawn and the laboratory characteristics i.e. High-density lipoproteins (HDL-cholesterol), total cholesterol, triglycerides, creatinine and serum glucose were measured through automated enzymatic method. C-reactive protein (CRP) was measured by an immunoturbidimetric assay on Roche/Hitachi cobas c systems. Estimated glomerular filtration rate (eGFR) was calculated by the CKD-EPI equation using serum creatine concentration, age and sex data, expressed as a single equation:

$${\text{eGFR}} = 141 \times \hbox{min} \left( {{\text{Scr}}/\upkappa, 1} \right)^{{\alpha}} \times \hbox{max} \left( {{\text{Scr}}/\upkappa, 1} \right)^{ - 1.209} \times 0.993^{Age} \times 1.018\left[ {if\;female} \right] \times 1.159\left[ {if\;black} \right]$$

where: Scr is serum creatinine in µmol/L, κ is 61.9 for females and 79.6 for males, α is − 0.329 for females and − 0.411 for males, min indicates the minimum of Scr/κ or 1, and max indicates the maximum of Scr/κ or 1.

Coffee consumption data was assessed from home interview in RS-I and RS-II and from a validated semiquantitative food frequency questionnaire at the Rotterdam Study center interviewed by a trained dietician in RS-III and expressed in g/day as a continuous variable. Grams of coffee consumed was calculated from frequency (expressed in cups/day), and one cup equals 150 g [26].

The total number of medication types used was calculated from the medication home interview taken by trained research assistants. During the home interview, participants presented all the medication they used in the past week. Data were checked for inconsistencies by the pharmaco-epidemiology group [27].

### Measurement of SAF

SAF was assessed later with a median time interval of 14.9[5.6–15.3] (mean time interval = 11.5) years after the 25(OH)D_3_ measurement in RS-I 6th follow-up (15.8 years), RS-II 4th follow-up (15.0 years) and R-III 2nd follow-up (5.5 years). The AGE Reader™ (DiagnOptics B.V., Groningen, The Netherlands) was used to determine SAF non-invasively, which is a marker for the amount of AGEs present in the skin. The mechanism is based on the fluorescent property of AGEs [28]. Meerwaldt and colleagues validated the SAF for AGEs (pentosidine, carboxymethyl-lysine (CML) and carboxyethyl-lysine (CEL)) accumulation in skin biopsies of the same site as where SAF was measured [29]. Around 4 cm^2^ of the skin was illuminated with an excitation light with a peak around 370 nm (between 300 and 420 nm) and guarded against surrounding light. The emission spectrum of AGEs (420–600 nm) is measured by the AGE Reader. SAF is calculated based on the ratio of excitation and reflected light, expressed in arbitrary units (A.U.), by AGE Reader software (version 2.3.0) using validated algorithm that accounts for skin color with a UV reflectance percentage (R%) of 6–10%. Participants with a mean skin reflectance of 6% or lower were automatically excluded by the AGE Reader™.

The dominant forearm of participants was placed on the device for three consecutive measurements of SAF. They were asked to not use skin creams before the measurement. Mean of three measurements was calculated to achieve accurate SAF value. Extreme value in triple measurements was identified and excluded through a combination of Grubbs’ test and value outside of the mean ± 4 SD scope. In this case, SAF was calculated as the mean of remaining 2 measurements.

### Statistical analysis

#### Statistical methods

Statistical analyses were performed through SPSS (version 21.0). Descriptive statistics described the parameters of the study population. Normality was determined by the use of histograms and Q–Q plots. Depending on the normal or non-normal distribution, data is represented as respectively mean (± SD) or median (interquartile range, IQR). Means of continuous variables between two groups were compared via the use of Mann–Whitney U-test when a non-normal distribution was assumed or independent samples T test when the variable was normally distributed. χ^2^ test was adopted to compare the means of categorical variables.

The association of 25(OH)D_3_ with SAF was analyzed via linear regression models. Simple linear regression analyses were used to study the crude association between a certain covariate and SAF. Multiple linear regression analyses were conducted with SAF being the outcome, whilst adjusting for covariates that may confound the association or potential risk factors for high SAF (model 1 adjusted for age, sex and RS subcohorts, data not shown; model 2 additionally adjusted for BMI, smoking status, DM status, eGFR, coffee intake and medication numbers). Multicollinearity was assessed by tolerance tests with a tolerance level of 0.40 considered threshold. Heteroscedasticity was determined by plotting the linear regression residuals and the predicted outcome values.

Two-way interactions between age, sex, RS subcohorts, smoking, diabetes, medication numbers, eGFR, coffee intake and 25(OH)D_3_ were checked by adding interaction terms into the linear model 2 because they were suspected to modify the association between 25(OH)D_3_ and SAF. A *p* value less than 0.1 was considered statistically significant.

Subgroup analysis was conducted to see if the association show disproportionate effects in predefined strata (diabetes or not, smoking status, sexes, RS subcohorts).

There were missing values in covariates BMI, eGFR, smoking, medication, diabetes status and coffee intake. To reduce potential bias caused by including only participants with complete information and exploit the information in incomplete record participants, we performed multiple imputation of missing data [30]. Missing data mechanism was distinguished as missing at random (MAR) from missing completely at random (MCAR) through Little’s test and t-test. Missing values were imputed by multiple imputations to maximize the sample size to 2746 participants. Predictive mean matching (PMM) method was used for multiple imputation of continuous variables to avoid implausible imputed values; for example it can be applied to non-normal distributed variables to avoid negative imputed values. Logistic regression was used for categorical variables. Several parameters were included in the multiple imputation procedure to make the missing at random assumption plausible. 20 imputations were obtained through 20 iterations. Sensitivity analysis was conducted to evaluate the imputation process and to see if the association between 25(OH)D_3_ and SAF remain consistent before and after multiple imputation. Details on the variables imputed are shown in Online Resource 1.

#### Covariates which may influence the association between 25(OH)D_3_ and SAF

We identified the potential risk factors for high SAF and confounders of the association between 25(OH)D_3_ and SAF at baseline based on a literature review and biological significance. SAF is known to increase with age and BMI [31], males show higher SAF than females [32]. The accumulation of AGEs is accelerated in diabetes mellitus (DM) [33] due to e.g., hyperglycemia [11]. Participants with a lower kidney filtration rate (indicated by eGFR) have an accelerated AGEs accumulation because of lower clearance rate through the kidney. Smokers have higher SAF [32]. Coffee intake was reported to be positively associated with SAF [32]. As an indicator of individual health status, total number of medication administrated by each subject may be a covariate. Cholesterol and C-reactive protein (CRP) were potential risk factors since AGEs were accelerated formed under hyperlipidemia [34] and inflammation environment (indicated by CRP). RS subcohorts were included in multiple linear regression models coded as dummy variables to account for potential subcohort effects. The time interval between 25(OH)D_3_ and SAF measurements varies and shares a strong collinearity with RS subcohorts, thus RS subcohorts was included in the models instead of time interval in order to keep additional subcohorts information. The associations of the above covariates with SAF were examined through simple linear regression and they were further adjusted in multiple linear regression models.

## Results

### Descriptives

Clinical and lifestyle characteristics of the total population, diabetic and non-diabetic population are shown in Table 1. Mean (± SD) SAF value was 2.40 (± 0.49) A.U. and mean (± SD) serum 25(OH)D_3_ concentration was 64.33 (± 26.92) nmol/L. 44.0% of the participants was male and 56.0% female. SAF was higher in diabetic ((2.61 ± 0.51) A.U.) than non-diabetic participants ((2.38 ± 0.49) A.U.). Compared with the non-diabetic group, participants with type 2 diabetes were older, more often male, had higher BMI, and higher fasting serum glucose. Furthermore, diabetic participants had lower values of serum 25(OH)D_3_, total cholesterol, HDL cholesterol and more types of medication use.

**Table 1:** Demographic and clinical characteristics in total population and stratified by diabetic status^a^

Parameters	Total population	Non-diabetic group	Diabetic group
N	2388	2206 (92.4%)	182 (7.6%)
Age (years)	61.19 ± 6.03	61.11 ± 6.02	62.19 ± 6.07
*Sex*			
Male/n (%)	1050 (44.0%)	951 (43.1%)	99 (54.4%)
Female/n (%)	1338 (56.0%)	1255 (56.9%)	83 (45.6%)
BMI (kg/m^2^)	27.15 ± 4.03	26.92 ± 3.89	29.95 ± 4.60
*Rotterdam study subcohort*			
RS I	606 (25.4%)	563 (25.5%)	43 (23.6%)
RS II	912 (38.2%)	833 (37.8%)	79 (43.4%)
RS III	870 (36.4%)	810 (36.7%)	60 (33.0%)
SAF (A. U.)	2.40 ± 0.49	2.38 ± 0.49	2.61 ± 0.51
25(OH)D_3_ (nmol/l)	64.33 ± 26.92	65.11 ± 27.06	54.87 ± 23.13
Total cholesterol (mmol/L)	5.74 ± 1.00	5.77 ± 0.99	5.41 ± 1.12
HDL cholesterol (mmol/L)	1.42 ± 0.39	1.44 ± 0.39	1.22 ± 0.34
eGFR (mL/min per 1.73 m^2^)	84.10 (73.78-93.78)	83.79 (73.58-93.70)	89.04 (77.06-95.82)
*Smoking status*, *n* (%)			
Never smokers	735 (30.8%)	688 (31.2%)	47 (25.8%)
Ex-smokers	1185 (49.6%)	1083 (49.1%)	102 (56.0%)
Current smokers	468 (19.6%)	435(19.7%)	33 (18.1%)
Serum fasting glucose (mmol/L)	5.50 (5.10–5.90)	5.40 (5.10–5.80)	7.60 (7.10–9.025)
Medication number	1 (0–3)	1 (0–3)	2 (1–4.25)
Coffee intake (g/d)	500.00 (375.00–638.39)	500.00 (375.00–638.39)	500.00 (375.00–638.39)
C-reactive protein (mg/mL)^b^	1.20 (0.49–2.77)	1.10 (0.41–2.62)	1.85 (0.70, 3.53)
Time interval between 25(OH)D_3_ and SAF measurement (years)	14.9 (5.6–15.3)	14.9 (5.6–15.3)	14.8 (5.6–15.3)

*SAF* skin auto-fluorescence; *BMI* body mass index; *25(OH)D*_*3*_ 25-Hydroxyvitamin D_3_; *HDL* high-density lipoproteins; *eGFR* estimated glomerular filtration rate calculated by the CKD-EPI

^a^Normally distributed data are expressed as mean ± standard deviation, non-normal distributed as median (interquartile range) and in the case of nominal variables as n (%)

^b^n = 9 participants had missing C-reactive protein information

### The association between 25(OH)D_3_ and SAF

The crude association between parameters of interest (age, sex, BMI, smoking status, 25(OH)D_3_, diabetes status, eGFR, RS subcohorts, sum of medication used during home interview, coffee intake, total cholesterol, CRP and time interval) and SAF investigated through simple linear regression is shown in Table 2. Higher SAF was significantly related to lower 25(OH)D_3_, lower eGFR, male sex, higher age, BMI and CRP, more coffee consumption, current smoker, diabetes, use of more medications, and longer time interval between measurements. The crude correlation coefficient between 25(OH)D_3_ and SAF was − 0.105 (*p* = 2.98 × 10^−7^), while after adjustment for age, sex and RS subcohorts, the partial correlation coefficient was − 0.147 (*p* = 5.11 × 10^−13^). Online Resource 4 shows the crude scatterplot and correlation between 25(OH)D_3_ and SAF and Online Resource 5 shows the scatter plot and partial correlation between 25(OH)D_3_ and SAF after age, sex and RS subcohorts adjustment.

**Table 2:** Simple linear regression and multiple linear regression results between covariates and SAF

Parameter	Simple linear regression^a^				Multiple linear regression^b^			
	Unstandardized coefficient B [95% CI]	Standardized coefficient β	Explained variance (%)^c^	*P* value	Unstandardized coefficient B [95% CI]	Standardized coefficient β	Explained variance (%)^d^	*P* value
Age	0.020 [0.017, 0.023]	0.248	6.2	< 0.0001	0.017 [0.013, 0.022]	0.213	2.3	< 0.0001
Sex^e^	− 0.208 [− 0.246, − 0.169]	− 0.209	4.4	< 0.0001	− 0.197 [− 0.235, − 0.159]	− 0.199	3.6	< 0.0001
BMI	0.009 [0.004, 0.014]	0.072	0.5	< 0.0001	0.005 [0.001, 0.010]	0.044	0.2	0.022
Smoking status			2.7				2.2	
Current smoker^f^	0.162 [0.113, 0.212]	0.131	–	< 0.0001	0.208 [0.155, 0.262]	0.168	–	< 0.0001
Ex-smoker^f^	0.022 [− 0.017, 0.062]	0.023	–	0.268	0.039 [− 0.003, 0.082]	0.040	–	0.067
25(OH)D_3_ (nmol/L)	− 0.002 [− 0.003, − 0.001]	− 0.105	1.1	< 0.0001	− 0.002 [− 0.003, − 0.002]	− 0.125	1.5	< 0.0001
DM status^g^	0.220 [0.146, 0.294]	0.119	1.4	< 0.0001	0.112 [0.042, 0.182]	0.060	0.3	0.002
eGFR (mL/min per 1.73 m^2^)	− 0.004 [− 0.006, − 0.003]	− 0.123	1.5	< 0.0001	− 0.001 [− 0.003, 3.06 × 10^−4^]	− 0.035	0.1	0.115
RS subcohorts			4.8				1.0	
RS-I^h^	0.143 [0.098, 0.188]	0.126	–	< 0.0001	0.096 [0.038, 0.155]	0.085	–	0.001
RS-II^h^	0.103 [0.063, 0.144]	0.102	–	< 0.0001	0.128[0.083, 0.174]	0.127	–	< 0.0001
Medication number	0.020 [0.009, 0.031]	0.074	0.5	< 0.0001	0.022 [0.012, 0.032]	0.082	0.6	< 0.0001
Coffee intake (g/d)	2.71 × 10^−4^ [2.01 × 10^−4^, 3.41 × 10^−4^]	0.153	2.3	< 0.0001	1.74 × 10^−4^ [1.04 × 10^−4^, 2.44 × 10^−4^]	0.098	0.8	< 0.0001
Cholesterol	− 0.019 [− 0.038, 0.001]	− 0.038	0.1	0.062	–	–	–	–
C-reactive protein	0.007 [0.002, 0.012]	0.054	0.3	0.009	–	–	–	–
Time interval	0.023 [0.019, 0.027]	0.220	4.9	< 0.0001	–	–	–	–

*SAF* skin auto-fluorescence; *BMI* body mass index; *25(OH)D*_*3*_ 25-hydroxyvitamin D_3_; *DM status* diabetes mellitus status; *eGFR* estimated glomerular filtration rate calculated by the CKD-EPI

^a^Outcome was SAF, without adjustment for covariates

^b^Model 2: SAF ~ Age + sex + BMI + smoking status + 25(OH)D_3_ + DM status + eGFR + coffee intake + medication numbers + RS subcohorts. N = 2388, R^2^ = 0.190, *p* < 0.0001. Collinearity test satisfied, no apparent collinearity

^c^Percentage of SAF variance explained by the variable in the simple linear regression model (%), equals R^2^ of the model × 100%

^d^Percentage of SAF variance explained by the variable in the multiple linear regression model (%), equals partial R^2^ of the variable × 100%

^e^Reference group is male participants

^f^Reference group is non-smoking participants

^g^Reference group is non-diabetic participants

^h^Reference group is participants from RS-III

In the multiple linear regression model 2 (Table 2), serum 25(OH)D_3_ remained significantly inversely associated with SAF (B = − 0.002, 95% CI[− 0.003, − 0.002], standardized β = − 0.125,) after adjustment for all covariates. Covariates were still significantly associated with higher SAF as higher age, male sex, BMI, diabetes, current smoker, more coffee consumption. A positive association between medication number and SAF (B = 0.022, 95% CI[0.012, 0.032], standardized β = 0.082) was also found. When we adjusted for time interval instead of RS subcohorts in model 2, the association between 25(OH)D_3_ and SAF was similar. No heteroscedasticity and collinearity were detected. Variance of SAF explained by model 2 is 19.0%, while the variance of SAF explained by 25(OH)D_3_ in this model was 1.5%, ranking second to sex (3.6%), age (2.3%) and smoking (2.2%) and higher than that of diabetes status (0.3%) and eGFR (0.1%).

### Sensitivity and subgroup analysis

#### Multiple imputation for missing value

Comparison of the complete dataset and the imputed dataset is shown in Online Resource 1. After multiple imputations, average coffee consumption (g/day), but not the other imputed variables, deviated slightly from the complete record dataset (485.05 g/day vs. 496.07 g/day ± 280.17 g/day).

Results of the multiple linear regression model 2 with imputed data in n = 2746 participants are presented in Online Resource 2. The coefficient of the association between 25(OH)D_3_ and SAF remained the same after multiple imputations (n = 2746, B = − 0.002, 95% CI [− 0.003, − 0.002], *p* < 0.0001) with that before multiple imputations (n = 2388, B = − 0.002, 95% CI [− 0.003, − 0.002], *p* < 0.0001). eGFR became significantly associated with SAF after multiple imputation. A similar association between 25(OH)D_3_ and SAF was also found (n = 358, B = − 0.003, 95% CI [− 0.004, − 0.001], *p* < 0.0001) after multiple imputation in the participants with incomplete data.

#### Subgroup analysis

Results of subgroup analysis is summarized in Online Resource 3. No significant two-way interaction was found between diabetes status, RS subcohorts, smoking status, sex and 25(OH)D_3_. Yet subgroup analysis was still conducted to evaluate if the association was consistent or disproportionate effects existed among subgroups of participants.

The association between 25(OH)D_3_ and SAF remained consistent among subgroups. There were 182 diabetic participants out of 2388 participants. The coefficient of serum 25(OH)D_3_ concentration on SAF was B = − 0.004 (95% CI [− 0.008, − 0.001], standardized coefficient β = − 0.192) in diabetics and B = − 0.002 (95% CI [− 0.003, − 0.002], standardized coefficient β = − 0.122) in non-diabetics. When analyzing RS subcohorts separately, it was noted that model 2 explained as much as 22.1% variance of SAF in RS-III, versus 13.4% and 13.6% in RS-I and RS-II. The variance of SAF explained by 25(OH)D_3_ alone in model 2 was 1.8%, 0.8%, and 3.4% in RS I, II and III respectively. The time interval between 25(OH)D_3_ and SAF measurements was the shortest in RS-III. 25(OH)D_3_ showed a consistent association in males and females, while the variation in SAF explained by model 2 was 19.7% in males and 12.9% in females.

## Discussion

Because vitamin D may prevent AGEs accumulation through its anti-inflammatory properties, we studied in a large prospective cohort (the Rotterdam Study) whether serum 25(OH)D_3_ concentration at baseline was associated with AGEs in the skin assessed by SAF at follow-up. There was a very consistent and statistically significant inverse association in all 3 subcohorts of RS, independently of other risk factors and potential confounders. Previously identified factors associated with high SAF, including higher age and BMI, male sex, diabetes, smoking and decreased kidney function (eGFR) [32, 35, 36], were also found to be significantly associated with high SAF in our study. We also confirmed recent findings of a relation between SAF and coffee consumption [35]. A novel association was found between the number of medications used and SAF, potentially indicating an association between impaired health status and higher skin AGEs. eGFR was significantly related to SAF in crude analysis, but not after controlling for age possibly because eGFR is correlated with age but it was significant in the multiple linear regression models in the imputed dataset. Serum cholesterol and CRP were no longer significantly associated with SAF in the multiple linear regression analysis so they were not included in the full model.

The inverse association between 25(OH)D_3_ and SAF was present despite a long median time interval of 14.9 years between the two measurements, which may be explained by the long half-life time of 14.8 (95% CI[9.4–22.3]) years of skin collagen [3]. The association was consistent in three subcohorts and the variance of SAF explained by 25(OH)D_3_ was highest in RS III, possibly related to the shorter time interval.

A few earlier cross-sectional studies on the relationship between 25(OH)D_3_ and SAF in smaller study populations showed inconsistent results. In 276 patients with type 1 and type 2 diabetes and 121 non-diabetic controls no association was observed [20]. Another cross-sectional study in 119 healthy participants and 27 hypertensive patients also reported no association [37]. In a recent preliminary report of 245 type 2 diabetes patients treated with lifestyle advice, metformin and/or sulphonylurea-derivatives, there was a significant inverse relationship shown between 25(OH)D_3_ and SAF independently of age, season, diabetes duration and renal function [21], consistent with our findings. Our data extend this association to non-diabetics and the general middle-aged and elderly population.

The observed association between 25(OH)D_3_ and SAF was highly significant in the multiple linear regression model (*p* = 6.89 × 10^−11^), and very consistent in the three RS subcohorts but the correlation was not very strong, explaining 1.5% of the variation. Much of the intra-individual variation in SAF has not yet been explained. Given the relatively small correlation coefficient we cannot exclude the possibility that the observed association may be related to residual confounding. However, it is of interest that in our study in the multiple linear regression analyses 25(OH)D_3_ levels explained more of the variance in SAF (1.5%) than type 2 diabetes (0.3%) or impaired kidney function (0.1%), which are considered well-known predictors of AGEs accumulation apart from age and sex [11, 38]. As participants from RS are densely phenotyped, we were able to take into consideration many potential confounders and the large sample size enabled good power to detect potential confounding effects.

There are potential explanations for the inverse relation between 25(OH)D_3_ and SAF. Vitamin D and its analogs may have an effect in preventing AGEs formation or accumulation. Salum et al. [18] recently found that oral administration of cholecalciferol led to a decrease of AGEs in the aortic wall of diabetic rats. Cholecalciferol and calcitriol prevented protein glycation in vitro [39]. Calcitriol may indirectly help with the clearance of AGEs through the kidney by protecting kidney structural integrity [40]. Vitamin D plays a role in alleviating oxidative stress [41] and inflammation response [42], which are the preferred environment for Millard reaction and AGEs formation.

Vitamin D may also counteract the deleterious effects of AGEs, which may indirectly reduce new AGEs formation. AGEs can interact with its receptor RAGE and activate the NF-κB pathway, invoking oxidative stress and inflammation [43]. Vitamin D can attenuate the activation of this pathway through vitamin D receptor, since the receptor can bind to IKKβ protein and block NF-κB activity [44]. Vitamin D was also observed to reduce the expression of RAGE [45]. In addition, sRAGE was shown to be increased by vitamin D supplements in women with polycystic ovary syndrome [19]. It acts as a decoy receptor by binding circulating AGEs, which leads to their clearance and prevents activation of NF-κB pathway and its ensuing harmful effects [46]. Positive effects of vitamin D are also supported by a previous in vitro study where calcitriol mitigated the deleterious effect of AGEs on endothelial cells [47]. Together with our results, these studies indicate a protective role of vitamin D in the accumulation and the detrimental effects of AGEs.

Another explanation for the association of vitamin D with SAF could be that skin AGEs may prevent the conversion of provitamin D into vitamin D in the skin by preventing vitamin D_3_ from being absorbed into the blood or altering the extracellular matrix, blocking photosynthesis.

Strengths of our study includes the availability of a large and well-phenotyped population-based cohort study with a long time interval between baseline determination of 25(OH)D_3_ and follow-up measurements of SAF. However, there are also weaknesses. SAF gives a non-perfect estimation of the amount of AGEs in the body. The AGE-Reader does not measure non-fluorescent AGEs, which also contribute to the total body AGE pool. Fluorescent components other than AGEs may influence SAF. There can also be local differences in AGE accumulation because of differences in turnover rate of affected proteins [3]. We cannot make conclusions about the causality of the inverse association between 25(OH)D_3_ and SAF. Also, we are not able yet to study a relation between SAF and incident diseases and a potential interaction with vitamin D because SAF was introduced in the RS recently. Serial measurements of vitamin D and SAF are not available. As discussed above, we cannot exclude the possibility of residual confounding. Also, we cannot exclude the possibility that our results are influenced by selective survival as there is long-term follow up in our study and elderly people and those with impaired health at the time of vitamin D measurement are most likely to drop out of the study.

In conclusion, serum 25(OH)D_3_ concentration measured at baseline was inversely associated with SAF level measured years later but the causality of this relation is yet unknown. Possible future research could investigate the changes in 25(OH)D_3_ after UV-exposure in participants with different AGEs levels. Also, studies are needed to test whether vitamin D levels or intake may modify associations of AGEs with incident diseases. Improving vitamin D status may lower AGE formation. Currently, some substances have been identified that may either decrease AGE formation, such as various plant polyphenols or may reduce the deleterious effects of AGEs such as cross-link breakers [48]. There are several potential AGEs lowering medications under investigation [49]. More insight into the role of AGEs and its relationship with vitamin D might provide new opportunities for the prevention or treatment of age-related diseases.

## Electronic supplementary material

Below is the link to the electronic supplementary material.

Supplementary material 1 (DOCX 145 kb)

## Abbreviations

AGEs: Advanced glycation end products
SAF: Skin autofluorescence
25(OH)D_3_: 25-hydroxyvitamin D_3_
RS: Rotterdam Study
eGFR: Estimated glomerular filtration rate
BMI: Body mass index
HDL: High-density lipoproteins
CRP: C-reactive protein
RAGE: Receptor for AGEs
NF-κB: Nuclear factor kappa-light-chain-enhancer of activated B cells
sRAGE: Soluble RAGE
A. U.: Arbitrary units
CML: Carboxymethyl-lysine
CEL: Carboxyethyl-lysine
MAR: Missing at random
MCAR: Missing completely at random
PMM: Predictive mean matching
DM: Diabetes mellitus
SD: Standard deviation
sVAP-1: Soluble vascular adhesion protein-1
UV: Ultraviolet
