# Supplementary material for: Serum 25-hydroxyvitamin D3 is associated with advanced glycation end products (AGEs) measured as skin autofluorescence: The Rotterdam Study
Source: Eur J Epidemiol. 2018 Sep 25;34(1):67–77. doi: 10.1007/s10654-018-0444-2 (PMC6325991; doi:10.1007/s10654-018-0444-2)

**Online Resources 1-5: Supplementary tables**

**Article title:**

Serum 25-Hydroxyvitamin D_3_ Is Associated With Advanced Glycation End Products (AGEs) Measured as Skin Autofluorescence in The Rotterdam Study.

**Journal name:**

European Journal of Epidemiology

**Author names:**

Jinluan Chen, Daniel van der Duin, Natalia Campos-Obando, M. Arfan Ikram ,Tamar E. C. Nijsten, André G. Uitterlinden, M. Carola Zillikens^*^

**Corresponding author:**

M. Carola Zillikens, Department of Internal Medicine, Erasmus Medical Center, 's-Gravendijkwal 230, 3015CE, Rotterdam, the Netherlands, email: m.c.zillikens@erasmusmc.nl

**Online Resource 1:**

**Supplementary Table 1** Comparison of the dataset without missing value and the dataset completed with imputed values

| Parameter | Missing, N(%)^a^ | Completed^b^  (mean ± SD, or n (%)) | Multiple imputation^c^ (pooled mean, or pooled n (%)) |
| --- | --- | --- | --- |
| BMI (kg/m^2^) | 12(0.4) | 27.20 ± 4.12 | 27.19 |
| eGFR (mL/min per 1.73m^2^) | 13 (0.5) | 83.32 ± 13.80 | 83.33 |
| Smoking status |  |  |  |
| Never smoker | 15 (0.5) | 840 (30.8) | 845 (30.8) |
| Ex-smoker |  | 1355 (49.6) | 1363 (49.6) |
| Current smoker |  | 536 (19.6) | 539 (19.6) |
| Medication | 39 (1.4) | 1.83 ± 1.86 | 1.83 |
| DM status |  |  |  |
| Non-diabetics | 49 (1.8) | 2491 (92.4) | 2532 (92.2) |
| Diabetics |  | 206 (7.6) | 214 (7.8) |
| Coffee (g/d) | 296 (10.8) | 496.07 ± 280.17 | 485.05 |

Abbreviations: SD, standard deviation; BMI, body mass index; DM status, diabetes mellitus status; eGFR, estimated glomerular filtration rate calculated by the CKD-EPI.

^a^ Percentage of participants with missing values of covariates from n=2746 participants.

^b^ Sample size: n=2388, all the participants had complete data record.

^c^ Sample size: n=2746.

**Online Resource 2:**

**Supplementary Table 2** Multiple linear regression^a^ results in the imputed dataset

| Parameter | Unstandardized coefficient, B [95% CI] | P value (significance) |
| --- | --- | --- |
| Age | 0.017 [0.014, 0.021] | <0.0001 |
| Sex^b^ | -0.188 [-0.223, -0.153] | <0.0001 |
| BMI (kg/m^2^) | 0.006 [0.002, 0.010] | 0.006 |
| Smoking status |  |  |
| Current smoker^c^ | 0.210 [0.161, 0.260] | <0.0001 |
| Ex-smoker^c^ | 0.044 [0.006, 0.083] | 0.024 |
| 25(OH)D_3_ (nmol/l) | -0.002 [-0.003, -0.002] | <0.0001 |
| DM status^d^ | 0.095 [0.030, 0.160] | 0.004 |
| eGFR(mL/min per 1.73m^2^) | -0.002 [-0.003, -6.80×10^-5^] | 0.040 |
| Coffee (g/d) | 1.91×10^-4^ [1.25×10^-4^, 2.58×10^-4^] | <0.0001 |
| Medication number | 0.020 [0.011, 0.030] | <0.0001 |
| RS subcohorts |  |  |
| RS-Ⅰ^e^ | 0.081 [0.029, 0.134] | 0.003 |
| RS-Ⅱ^e^ | 0.123 [0.082, 0.164] | <0.0001 |

Abbreviation: BMI, body mass index; 25(OH)D_3_, 25-Hydroxyvitamin D_3_; DM status: diabetes mellitus status; eGFR: estimated glomerular filtration rate calculated by the CKD-EPI.

^a^ Model information: SAF ~ Age + sex + BMI + smoking status + 25(OH)D_3_ + DM status + eGFR + coffee intake + medication numbers + RS subcohorts. N=2746.

^b^ Reference comparison group is male participants;

^c^ Reference comparison group is non-smoking participants;

^d^ Reference comparison group is non-diabetic participants;

^e^ Reference comparison group is participants of RS-III.

**Online Resource 3:**

**Supplementary Table 3** The association between SAF and 25(OH)D_3_ in the sensitivity and subgroup analysis

| Subgroups | Model parameters | | |
| --- | --- | --- | --- |
|  | Coefficient B (95% CI), β^a^ | P (Significance) | Variance explained^b^ |
| Complete record group^c^, n=2388 | -0.002[-0.003, -0.002], -0.125 | <0.0001 | 1.5% / 19.0% |
| Multiple imputation^c^, n=2746 | -0.002[-0.003, -0.002], -- | <0.0001 | -- |
| Imputed group^c*^, n=358 | -0.003[-0.004, -0.001], -- | <0.0001 | -- |
| DM status^d^ |  |  |  |
| Non-diabetics | -0.002[-0.003, -0.002], -0.122 | <0.0001 | 1.4% / 18.0% |
| diabetics | -0.004[-0.008, -0.001], -0.192 | 0.011 | 3.2% / 18.4% |
| RS subcohorts^e^ |  |  |  |
| RS Ⅰ | -0.003[-0.005, -0.001], -0.146 | <0.0001 | 1.8% / 13.4% |
| RS Ⅱ | -0.002[-0.003, -0.001], -0.089 | 0.005 | 0.8% / 13.6% |
| RS Ⅲ | -0.003[-0.004, -0.002], -0.190 | <0.0001 | 3.4% / 22.1% |
| Smoking status^f^ |  |  |  |
| Never smoker | -0.002[-0.003, -0.001], -0.115 | 0.001 | 1.2% / 16.7% |
| Ex-smoker | -0.002[-0.003, -4.75×10^-4^], -0.114 | <0.0001 | 1.2% / 18.9% |
| Current smoker | -0.003[-0.005, -0.002], -0.173 | <0.0001 | 2.9% / 15.2% |
| Sex^g^ |  |  |  |
| Male | -0.003[-0.004, -0.002], -0.154 | <0.0001 | 2.2% / 19.7% |
| Female | -0.002[-0.003, -0.001], -0.120 | <0.0001 | 1.3% / 12.9% |

Abbreviation: SAF, skin auto-fluorescence; 25(OH)D_3_, 25-Hydroxyvitamin D_3_; DM status: diabetes mellitus status.

^a^ Values are unstandardized coefficient (B) of 25(OH)D_3_ and standardized coefficient (β) of 25(OH)D_3_ in the linear regression models.

^b^ SAF variance explained by 25(OH)D_3_/ SAF variance explained by the model.

^*^ The dataset which only included participants with incomplete data record, where the missing values were filled by multiple imputation method.

Models information:

^c^ SAF ~ Age + sex + BMI + smoking status + 25(OH)D_3_ + DM status + eGFR + coffee intake + medication numbers + RS subcohorts.

^d^ SAF ~ Age + sex + BMI + smoking status + 25(OH)D_3_ + eGFR + coffee intake + medication numbers + RS subcohorts, stratified by DM status.

^e^ SAF ~ Age + sex + BMI + smoking status + 25(OH)D_3_ + DM status + eGFR + coffee intake + medication numbers, stratified by RS subcohorts.

^f^ SAF ~ Age + sex + BMI + 25(OH)D_3_ + DM status + eGFR + coffee intake + medication numbers + RS subcohorts, stratified by smoking status.

^g^ SAF ~ Age + BMI + smoking status + 25(OH)D_3_ + DM status + eGFR + coffee intake + medication numbers + RS subcohorts, stratified by sex.

**Online Resource 4:**

**Supplementary Figure 1** Scatter plot of 25(OH)D_3_ and SAF in the study population


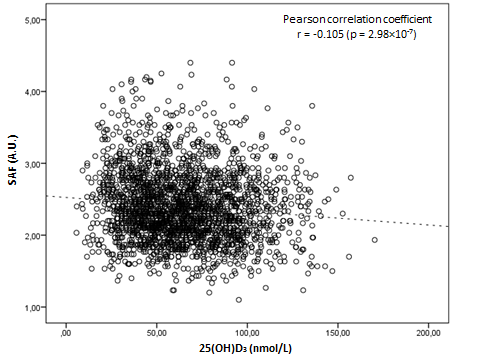


**Online Resource 5:**

**Supplementary Figure 2** Scatter plot of 25(OH)D_3_ and SAF after adjustment of age, sex and RS subcohorts


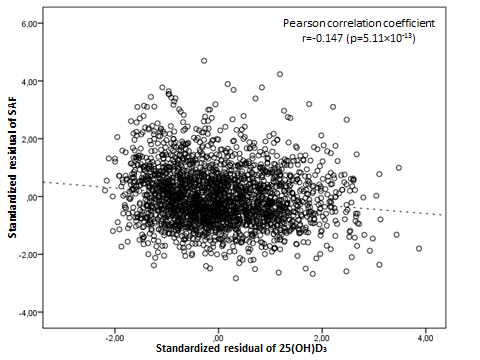

Supplement: Supplementary file 1 — Supplementary material 1 (DOCX 145 kb) [file 10654_2018_444_MOESM1_ESM.docx]
